# Zaluzanin C Alleviates Inflammation and Lipid Accumulation in Kupffer Cells and Hepatocytes by Regulating Mitochondrial ROS

**DOI:** 10.3390/molecules28227484

**Published:** 2023-11-08

**Authors:** Ji-Won Jung, Feng Wang, Ayman Turk, Jeong-Su Park, Hwan Ma, Yuanqiang Ma, Hye-Rin Noh, Guoyan Sui, Dong-Su Shin, Mi-Kyeong Lee, Yoon Seok Roh

**Affiliations:** College of Pharmacy, Chungbuk National University, Cheongju 28160, Republic of Korea; jw69884869@gmail.com (J.-W.J.); wang979253414@gmail.com (F.W.); aymanturk@chungbuk.ac.kr (A.T.); 6318js@gmail.com (J.-S.P.); akghks5065@gmail.com (H.M.); yuangqiangma123@gmail.com (Y.M.); sgf951110@gmail.com (H.-R.N.); suiguoyan1996@gmail.com (G.S.); dongss1429@gmail.com (D.-S.S.)

**Keywords:** NAFLD, Zaluzanin C, mtROS, inflammation, lipid accumulation

## Abstract

Zaluzanin C (ZC), a sesquiterpene lactone isolated from *Laurus nobilis* L., has been reported to have anti-inflammatory and antioxidant effects. However, the mechanistic role of ZC in its protective effects in Kupffer cells and hepatocytes has not been elucidated. The purpose of this study was to elucidate the efficacy and mechanism of action of ZC in Kupffer cells and hepatocytes. ZC inhibited LPS-induced mitochondrial ROS (mtROS) production and subsequent mtROS-mediated NF-κB activity in Kupffer cells (KCs). ZC reduced mRNA levels of pro-inflammatory cytokines (Il1b and Tnfa) and chemokines (Ccl2, Ccl3, Ccl4, Cxcl2 and Cxcl9). Tumor necrosis factor (TNF)-α-induced hepatocyte mtROS production was inhibited by ZC. ZC was effective in alleviating mtROS-mediated mitochondrial dysfunction. ZC enhanced mitophagy and increased mRNA levels of fatty acid oxidation genes (Pparα, Cpt1, Acadm and Hadha) and mitochondrial biosynthetic factors (Pgc1α, Tfam, Nrf1 and Nrf2) in hepatocytes. ZC has proven its anti-lipid effect by improving lipid accumulation in hepatocytes by enhancing mitochondrial function to facilitate lipid metabolism. Therefore, our study suggests that ZC may be an effective compound for hepatoprotection by suppressing inflammation and lipid accumulation through regulating mtROS.

## 1. Introduction

Non-alcoholic fatty liver disease (NAFLD) represents a range of liver conditions, spanning from basic steatosis to hepatitis, fibrosis, cirrhosis, and hepatocellular carcinoma [1,2,3,4]. The global prevalence of NAFLD is estimated to be 25.24% [5]. To date, there are no Food and Drug Administration (FDA)-approved treatments for NAFLD/NASH. Kupffer cell (KC) activation is believed to be crucial in promoting intrahepatic lipid accumulation during NAFLD. Cytokines released due to the inflammatory response of KCs inflict hepatocyte damage and foster lipid accumulation [6,7]. The persistent lipid accumulation in hepatocytes, along with chronic inflammatory responses, can culminate in the progression of NASH beyond NAFLD [8]. Consequently, it is imperative to conduct further research for the development of novel therapeutic approaches and target discovery for NAFLD.

Mitochondria, the principal energy-generating organelles, govern homeostasis and lipid metabolism [9]. mtROS have emerged as potential contributors to the pathogenesis of NAFLD [10]. LPS binding to TLR-4 receptors on KCs triggers mtROS generation, subsequently initiating an inflammatory response via NF-κB signaling activation [11]. Among the cytokines secreted by KCs during the inflammatory response, tumor necrosis factor (TNF)-α activates hepatocytes [12]. TNF-α induces mtROS in hepatocytes, which in turn induces mitochondrial dysfunction, inhibits fatty acid oxidation, and aids in lipid accumulation in hepatocytes [13,14,15]. Persistent and excessive mtROS production has been implicated in the inflammatory response and hepatic steatosis observed in both KCs and hepatocytes [16,17]. Based on these observations, there is a pressing need for further exploration of the mechanisms underlying mtROS in NAFLD.

ZC, a sesquiterpene lactone derived from the leaves of *Laurus nobilis* L., is known to have antioxidant, anti-inflammatory and anti-lipid effects, but the efficacy and mechanism of action in non-alcoholic fatty liver disease have not been elucidated [18,19,20]. Therefore, in this study aim to elucidate the effect of ZC on the mechanism of action of NAFLD by ameliorating inflammation and hepatic steatosis through an mtROS-modulating effect.

## 2. Results

### 2.1. ZC Inhibits LPS-Induced ROS Production in Immortalized Mouse Kupffer Cells (ImKCs)

**Figure 1:**
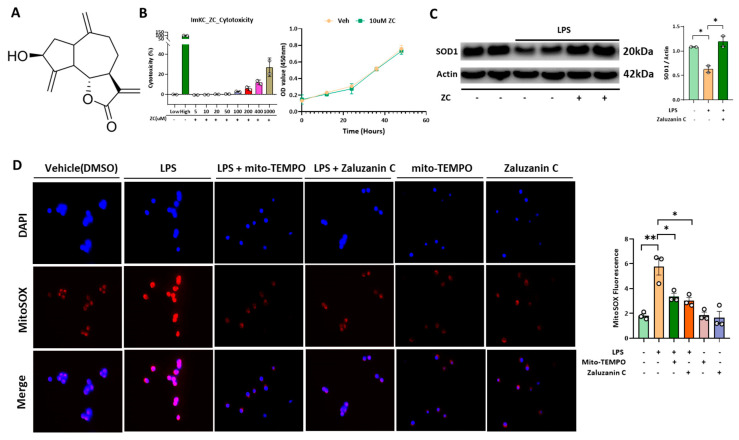
ZC inhibits LPS-induced ROS in Immortalized mouse kupffer cells (ImKC). (**A**) Structure of ZC. (**B**) Quantification of ZC cytotoxicity in ImKCs and cells counting assay with 10 μM ZC in ImKCs. Cytotoxicity assay was performed using the Quanti-LDH™ Cytotoxicity Assay Kit. ImKCs were measured using a fluorescence microscope and microplate reader. (**C**) Representative Western blotting analysis of SOD1. The concentration of LPS was 250 ng/mL, and for ZC, it was 10 μM. (**D**) MitoSOX 6 h after LPS (500 ng/mL), ZC (10 μM) and Mito-TEMPO (50 μM) treatment. The data are expressed as means ± sem. * *p* < 0.05, ** *p* < 0.01.

LPS binding to the TLR-4 receptor induces the generation of mtROS [21]. To determine the optimal concentration of ZC for study, we examined the cytotoxicity in ImKCs from 5 μM to 1000 μM (Figure 1B). ZC exhibited no toxicity and no growth inhibitory effect at concentrations up to 10 μM in ImKCs. Notably, ZC effectively reduced LPS-induced mtROS production (Figure 1C,D). This effect of ZC mirrored that of Mito-TEMPO, an mtROS inhibitor, in suppressing mtROS levels. The loss of SOD1, one of the components of superoxide dismutases (SODs), which is the basis of the oxidative stress defense system, causes oxidative DNA damage to cells and increases ROS levels [22]. This study demonstrated that ZC ameliorated the LPS-induced decline in SOD1 protein levels. Collectively, these results suggest that ZC preserves SOD1 and exerts antioxidant effects on ImKCs by improving LPS-mediated mtROS production.

### 2.2. ZC Ameliorates Inflammation by Inhibiting ROS-Induced NF-κB Signaling in Kupffer Cells

**Figure 2:**
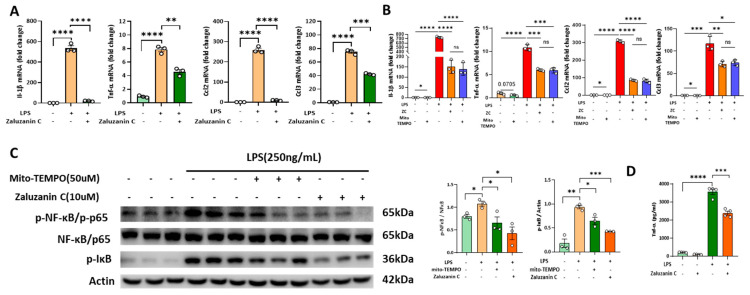
ZC has anti-inflammatory effects by inhibiting ROS-induced NF-κB signaling. (**A**) The mRNA expression of cytokines and chemokines treated with LPS (125 ng/mL) and ZC (10 μM) in ImKCs. (**B**) The mRNA expression of cytokines and chemokines treated with LPS (125 ng/mL), ZC (10 μM) and Mito-TEMPO (50 μM) in ImKCs. (**C**) Representative Western blotting analysis of p-NF-κB, NF-κB and p-IκB, Actin. The concentration of LPS was 250 ng/mL and ZC was 10 μM and Mito-TEMPO was 50 μM. (**D**) The protein level of TNF-α in ImKC lysates were determined using Enzyme-linked Immunosorbent Assay. Relative mRNA expression levels were normalized to mouse GAPDH levels. The data are expressed as means ± sem. ^ns^ *p* > 0.05, * *p* < 0.05, ** *p* < 0.01, *** *p* < 0.001, and **** *p* < 0.0001.

Elevated ROS production triggers NF-κB signaling activation, resulting in inflammation [23,24]. ZC reduced the mRNA expression levels of pro-inflammatory cytokines and chemokines, such as IL-1β, TNF-α, Ccl2, and Ccl3 (Figure 2A), which were initially elevated by LPS. In addition, to investigate whether ZC alleviates inflammatory response through inhibiting ROS production, Mito-TEMPO was used as a negative control, as shown in Figure 2B; ZC and Mito-TEMPO treatment exert similar inhibitory effect in LPS-induced inflammation. Furthermore, both ZC and Mito-TEMPO decreased the LPS-induced phosphorylation of NF-κB and IκB in ImKCs (Figure 2C). Mito-TEMPO inhibited NF-κB signaling, indicating that NF-κB signaling activation is mtROS-mediated. Moreover, ZC treatment induced the suppression of TNF-α production in ImKCs (Figure 2D). In conclusion, our data showed that ZC inhibits mtROS-mediated NF-κB signaling. In conclusion, our data provide evidence that ZC effectively inhibits mtROS-mediated NF-κB signaling. Moreover, ZC demonstrated the ability to counteract the LPS-induced upregulation of pro-inflammatory markers, suggesting its potent anti-inflammatory properties.

### 2.3. ZC Inhibits ROS Production in Hepatocytes Induced by TNF-α Secreted by ImKCs

**Figure 3:**
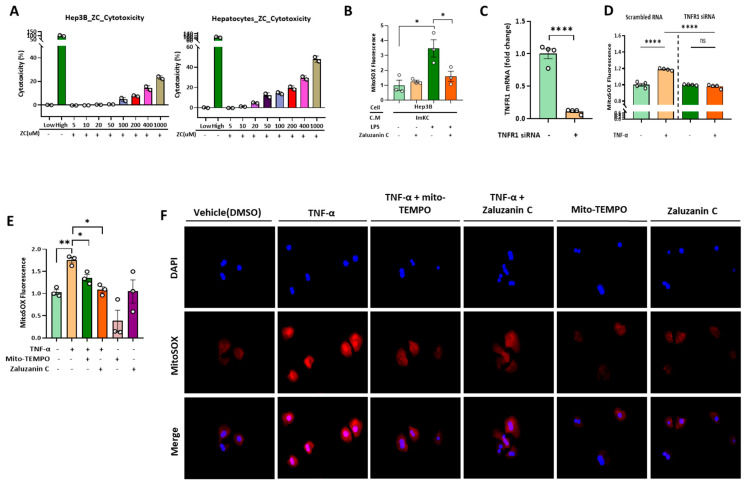
ZC inhibits ROS in hepatocytes induced by TNF-α secreted by Immortalized mouse kupffer cells (ImKCs). (**A**) Hep3B cells and primary hepatocytes were treated with different ZC concentrations (5 μM to 1000 μM) and cytotoxicity was quantified. (**B**) Mitochondrial superoxides (MitoSOXs) were measured in Hep3B cells treated with supernatant collected from Immortalized mouse kupffer cells (ImKCs). (**C**) mRNA expression of TNFR1 after TNFR1 siRNA transfection in primary hepatocytes. (**D**) Primary hepatocytes transfected with TNFR1 siRNA and scrambled RNA control (SC) were treated with TNF-α (100 ng/mL) for 24 h, and MitoSOXs were measured with a microplate reader. (**E**) Hep3B cells were measured using a microplate reader 24 h after treatment with TNF-α (100 ng/mL), ZC (10 μM) and Mito-TEMPO (50 μM). (**F**) Primary hepatocytes were observed by fluorescence microscopy 6 h after treatment with TNF-α (10 ng/mL), ZC (10 μM) and Mito-TEMPO (50 μM). Data are presented as mean ± sem. ^ns^ *p* > 0.05, * *p* < 0.05, ** *p* < 0.01, and **** *p* < 0.0001.

TNF-α, released during inflammatory responses, induces mtROS generation in hepatocytes [14]. To determine the optimal concentration of ZC for this study, we assessed its cytotoxicity in Hep3B cells and primary hepatocytes (Figure 3A). ZC exhibited no toxicity at concentrations up to 10 μM in Hep3B and primary hepatocytes. Remarkably, ZC inhibited mtROS production in hepatocytes activated with conditioned medium from ImKC (Figure 3B). TNFR1 siRNA treatment reduced the mRNA level of TNFR1 in primary hepatocytes (Figure 3C). This study found that TNFR1 knockdown decreased mtROS production (Figure 3D), suggesting that TNF-α binds to the TNFR1 receptor in primary hepatocytes and induces mtROS. Additionally, ZC and Mi-to-TEMPO reduced TNF-α-induced mtROS production in Hep3B and primary hepatocytes (Figure 3E,F). This effect of ZC was found to closely resemble that of mtROS inhibition upon Mito-TEMPO administration, a mtROS inhibitor. Collectively, these results suggest that ZC inhibits mtROS in Hep3B by regulating Kupffer cell activity. Additionally, it is evident that ZC inhibits TNF-α-induced mtROS production in Hep3B.

### 2.4. ZC Alleviates MtROS-Induced Mitochondrial Dysfunction in Hepatocytes

**Figure 4:**
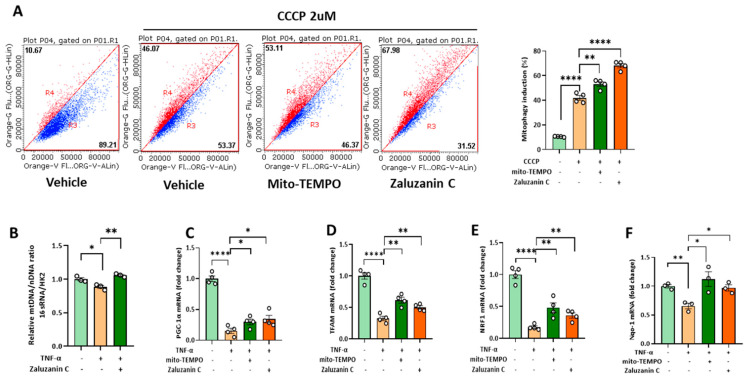
ZC alleviates mitochondrial dysfunction. (**A**) Mitophagy detection after ZC and Mito-TEMPO treated with for 24 h. ZC- and Mito-TEMPO-mediated mitophagy was quantified by Flow Cytometry with 2 μM carbonyl cyanide m-chlorophenylhydrazone (CCCP). (**B**) qPCR analysis in primary hepatocytes for mitochondrial DNA (16s) expression normalized to HK2. (**C**–**F**) mRNA expression of mitochondrial biogenesis factors treated with TNF-a (30 ng/mL) and ZC (10 μM) in Hep3B cells. The data are expressed as means ± sem. * *p* < 0.05, ** *p* < 0.01, and **** *p* < 0.0001.

The persistent accumulation of mtROS causes mitochondrial dysfunction [25]. This study focused on exploring the involvement of ZC in mitophagy, an essential mechanism responsible for removing damaged mitochondria [26] using the mt-Keima system, which is a specific fluorescent protein-based marker used for monitoring mitophagy, a process of selective autophagy involving the removal of damaged or dysfunctional mitochondria within cells [27]. According to our setting, when mitophagy occurs, the signal will increase in the R4 area as a percentage state, while R3 means there is no mitophagy induction area. Interestingly, treatment with ZC and Mito-TEMPO augmented CCCP-induced mitophagy (Figure 4A). Additionally, considering the effect of ZC on mitochondrial function, to understand the regulation of mitochondrial biogenesis, we assessed the PGC-1α-NRF1-TFAM pathway and mtDNA [28]. Our findings revealed a significant increase in mitochondrial DNA content, as indicated by the expression level of 16S rRNA encoding mitochondrial genes (Figure 4B). Moreover, ZC upregulated the expression levels of PGC-1α, TFAM, Nrf1, and Npo-1, key regulators of mitochondrial biogenesis, thereby exerting mitochondrial protective effects (Figure 4C–F).

### 2.5. ZC Ameliorates ROS-Induced Lipid Accumulation

**Figure 5:**
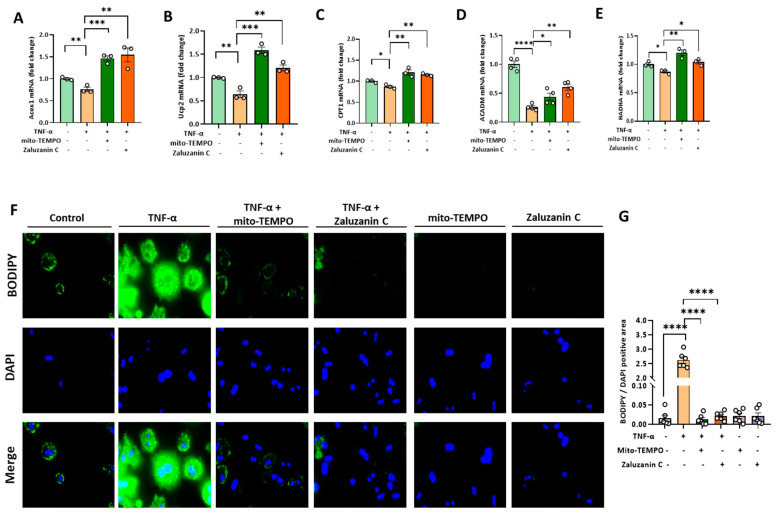
ZC improves ROS-induced lipid accumulation through β-oxidation activity. The mRNA levels of the beta-oxidation gene markers Acox1 (**A**), Ucp2 (**B**), CPT1 (**C**), ACADM (**D**) and HADHA (**E**) were measured by qRT-PCR in Hep3B cells. Hep3B cells were treated with TNF-α 30 ng/mL and ZC 10 μM. (**F**) BODIPY staining in primary hepatocytes. (**G**) Quantification of BODIPY/DAPI-positive areas. Relative mRNA expression levels were normalized to human actin levels. Data are presented as mean ± sem. * *p* < 0.05, ** *p* < 0.01, *** *p* < 0.001, and **** *p* < 0.0001.

TNF-α induces mtROS production in hepatocytes [13,15]. Elevated mtROS levels impair PPARα function and hinder PPARα-mediated β-oxidation processes [29,30]. Decreased β-oxidation processes hamper lipolysis in hepatocytes, leading to hepatic steatosis [31]. This study demonstrated that ZC exerts a potent anti-steatotic effect by upregulating the mRNA expression levels of β-oxidation markers (Ucp2, Acox1, CPT1, ACADM and HADHA) that were otherwise suppressed by TNF-α in Hep3B cells (Figure 5A–E). Notably, ZC and Mito-TEMPO ameliorated TNF-α-induced lipid accumulation in primary hepatocytes (Figure 5F,G). The findings indicate that Mito-TEMPO effectively hindered lipid accumulation and provide evidence for the involvement of mtROS in the process of lipid accumulation. Overall, our data demonstrated that ZC has an anti-steatotic effect via regulating β-oxidation. Furthermore, ZC ameliorates ROS-mediated lipid accumulation, suggesting its potential as a protective agent against hepatic steatosis.

## 3. Discussion

Recently, NAFLD research has been primarily centered on normalizing metabolism by targeting lipid accumulation and reducing inflammatory responses through the regulation of hepatic fatty acid synthesis and oxidation processes [32,33,34]. There is increasing evidence pointing to the significant role of mtROS in sustaining inflammation and fat ac-cumulation, underscoring the importance of mtROS modulation in NAFLD treatment [35,36]. The initial therapeutic approach for NAFLD through mtROS modulation involves reducing the inflammatory response by inhibiting mtROS-mediated NF-κB signaling in KCs [37]. Furthermore, TNF-α, which is secreted during inflammation, induces mtROS production in hepatocytes, leading to mitochondrial damage [38,39,40,41]. Impaired mitochondria subsequently compromise PPARα function, leading to a reduced beta-oxidation process [42]. Another therapeutic strategy for NAFLD involves promoting beta-oxidation to enhance lipid breakdown in hepatocytes [43].

ZC is known as a compound with excellent antioxidant and anti-inflammatory effects, but studies related to NAFLD have not yet been conducted [18,19]. This study found that ZC had a significant effect in inhibiting mtROS. Also, it elucidated the mechanism by which ZC exerts anti-inflammatory effects by inhibiting the mtROS-mediated NF-κB pathway and alleviates NAFLD through the modulation of mtROS-mediated lipid accumulation. ZC significantly reduced LPS-induced mtROS in Kupffer cells. This is similar with the reducing effect of mtROS confirmed using Mito-TEMPO, which is known as an inhibitor of mtROS. ZC decreased mRNA levels of inflammatory cytokines and chemokine factors increased by LPS. Also, it shows that ZC has anti-inflammatory effects by inhibiting activated NF-κB signaling through the generation of mtROS. Our study revealed that TNF-α induces mtROS by binding to the TNFR1 receptor in hepatocytes. mtROS was significantly increased when TNF-α was treated in primary hepatocytes using control siRNA, but there was no change in mtROS expression after TNF-α treatment in TNFR1 knockdown primary hepatocytes using TNFR1 siRNA. ZC significantly reduced TNF-α-induced mtROS in hepatocytes, similar with the mtROS-reducing effect of Mito-TEMPO. An excessive increase in mtROS causes mitochondrial dysfunction [25]. Indicators for identifying mitochondrial dysfunction include, for example, the damage and release of mitochondria DNA (mtDNA), and abnormal mitophagy [44,45]. In our study, ZC alleviated mitochondrial dysfunction by repairing mtDNA damaged by TNF-α and promoting the mitophagy effect induced by CCCP treatment using Mt-Keima system. Mt-Keima, while a valuable tool, has its limitations. Mt-Keima is designed to change its fluorescence properties based on the pH of the environment [26]. However, there can be some limitations due to partial spectral overlap in the excitation spectra of Keima in acidic environments. This spectral overlap can make it challenging to distinguish between the red signal originating from the mitochondria and the green signal from the lysosomes, leading to the observed orange color [27]. It is essential to be aware of such potential issues when using Mt-Keima and consider them when interpreting the results of experiments related to mitophagy. In addition, ZC increased the mRNA levels of beta-oxidation genes that were reduced by TNF-α. Furthermore, BODIPY staining analysis suggests that ZC inhibits mtROS-mediated lipid accumulation in hepatocytes and has anti-lipid effects.

Further studies are needed to demonstrate the effect of ZC on NAFLD. Although our study demonstrated the antioxidant, anti-inflammatory and anti-lipid effects of ZC on NAFLD through in vitro studies, more direct studies through in vivo studies are needed. The improvement effect of NAFLD can be confirmed through blood analysis (AST and ALT) and pathological tissue analysis, such as H&E and Oil red O staining, in a mouse model that induces obesity through FFD (Fast Food Diet) feeding [46,47]. In addition, the effect of ZC on liver fibrosis can be demonstrated by Sirius Red staining analysis and the measurement of mRNA levels of fibrosis-related genes [48,49,50]. The liver-fibrosis-improving effect of ZC may lead to the extension of NASH treatment.

## 4. Materials and Methods

### 4.1. Isolation of ZC

**Scheme 1:**
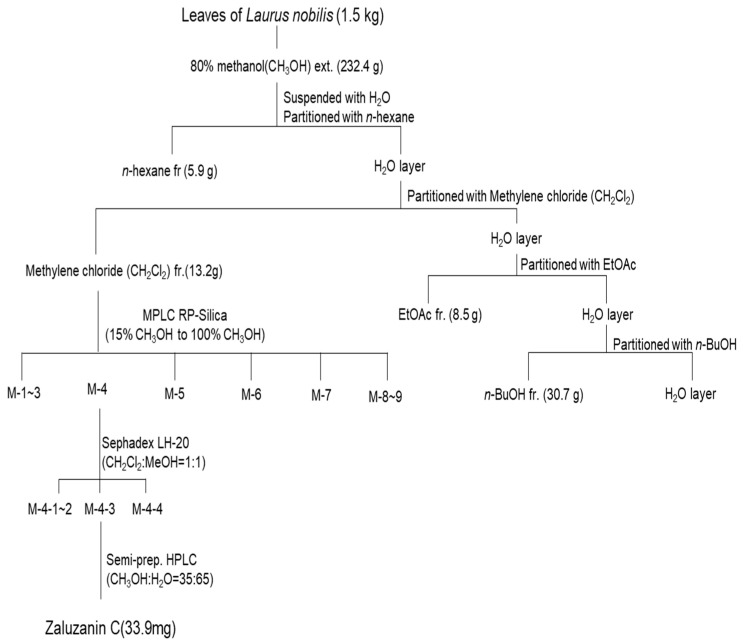
Isolation method of ZC from leaves of *L. nobilis*.

ZC was isolated as previously described using dried leaves of *Laurus nobilis* L. obtained from Lebanon in August 2014 (Figure 1A) [51]. A voucher specimen (CBNU-2014-LN) was deposited in the Herbarium of the College of Pharmacy, Chungbuk National University (Osong, Cheongju, Republic of Korea). Briefly, *L. nobilis* L. leaves (1.5 kg) were extracted with 80% methanol (CH_3_OH) to obtain a crude extract (232.4 g). The methanol extract was then suspended in H_2_O and partitioned sequentially with *n*-hexane, methylene chloride (CH_2_Cl_2_), ethyl acetate (EtOAc) and butanol (*n*-BuOH). The methylene chloride (CH_2_Cl_2_) fraction was subjected to MPLC RP-Silica (15% CH_3_OH to 100% CH_3_OH) to obtain nine fractions (M1 to M9). ZC was purified from M-4 by Sephadex LH-20 column chromatography (CH_2_Cl_2_:MeOH = 1:1) followed by semi-preparative HPLC (MeOH:H_2_O = 35:65) (Scheme 1).

### 4.2. Chemicals

Dulbecco’s Modified Eagle’s Medium (DMEM), Corning^®^, Glendale, CA, USA; M199 medium, Corning^®^, CA, USA; penicillin–streptomycin solution 100× (PS), Corning^®^, CA, USA; Recombinant Human TNF-α, BioLegend; fetal bovine serum (FBS), Corning^®^, Glendale, USA; Recombinant Mouse TNF-α, BioLegend; Mito-TEMPO, Santa Cruz Biotechnology, Inc., Dallas, TX, USA; Pierce™ BCA Protein Assay Kit, Thermo Scientific, Waltham, MA, USA; phosphorylated-IkBα, Cell Signaling Technology, Danvers, MA, USA; TB Green Premix Ex Taq II, TAKARA, Kusatsu, Japan; phosphorylated-NF-κB, Cell Signaling Technology, Danvers, MA, USA; lipopolysaccharide (LPS), Sigma-Aldrich, St. Louis, MO, USA; nuclear factor kappa-light-chain-enhancer of activated B cells, Abclonal, Woburn, MA, USA; superoxide dismutase 1, ABclonal; actin antibodies, Sigma-Aldrich, St. Louis, MO, USA.

### 4.3. Immortalized Mouse Kupffer Cells (ImKCs) and Hep3B Cell Culture

Immortalized mouse kupffer cells (ImKCs) were purchased from Applied Biological Materials Inc. (Richmond, BC, Canada), 10% FBS (fetal bovine serum) was added to Dulbecco’s Modified Eagle’s Medium (DMEM). Incubation occurred in an environment of 37 °C, 5% CO_2_. To evaluate the mtROS inhibitory effect of ZC, ImKCs (5 × 10^4^ cells/mL) were treated with lipopolysaccharide (LPS, 500 ng/mL), ZC (10 μM) and Mito-TEMPO (50 μM) for 6 h. In order to measure the protein level of the related gene, ImKCs (4 × 10^5^ cells/mL) were pre-treated with ZC (10 μM) for 5 h and then simultaneously treated with lipopolysaccharide (LPS, 250 ng/mL) for 1 h. To confirm the anti-inflammatory effect of ZC and to measure mRNA levels, ImKCs (2 × 10^5^ cells/mL) were treated with lipopolysaccharide (LPS, 125 ng/mL) and ZC (10 μM) for 6 h. Lipopolysaccharide (LPS, 250 ng/mL), ZC (10 μM), and Mito-TEMPO (50 μM) were treated in ImKCs (4 × 10^5^ cells/mL) for 6 h to measure the protein level of the related gene.

Hep3B cells were purchased from the American Type Culture Collection (ATCC) and cultured in Dulbecco’s Modified Eagle’s Medium (DMEM) supplemented with 10% fetal bovine serum (FBS) at 37 °C and 5% CO_2_. In order to evaluate the mtROS inhibitory efficacy of ZC, Hep3B (3 × 10^4^ cells/mL) cells were treated with tumor necrosis factor (TNF)-α (100 ng/mL), ZC (10 μM) and Mito-TEMPO (50 μM) for 24 h. To measure the anti-lipid effects and mRNA levels of ZC, Hep3B (2 × 10^5^ cells/mL) cells were treated with TNF-α (30 ng/mL), ZC (10 μM) and Mito-TEMPO (50 μM) for 24 h.

### 4.4. Primary Hepatocyte Cell Culture

Male C57BL/6 mice at 8 weeks of age were procured from Samtako Bio Korea, located in Osan, Korea. To isolate primary hepatocytes, the mice were first anesthetized using Zoletil (30 mg/kg, Virbac, Carros, France). Following anesthesia, a laparotomy was performed, and a 24 G catheter was inserted into the inferior vena cava (IVC). The liver was perfused with 30 mL of EGTA solution, which was maintained at a temperature of 39.5 °C. Perfusion was achieved using a Masterflex L/S easy-load II pump from Cole-Parmer Instrument Co. based in Vernon Hills, IL, USA. This step aimed to completely remove blood from the liver. Subsequently, the liver was perfused with an enzyme buffer containing collagenase type 1 (650 μg/mL) sourced from Worthington Biochemicals in Los Angeles, CA, USA, and collagenase P (50 μg/mL) obtained from Roche in Mannheim, Germany. After the perfusion was completed, the liver was removed, minced and filtered through a 100 μm filter. The filtered hepatocytes were washed twice with enzyme buffer and then resuspended in complete M199 medium (Corning, NY, USA). To measure mtROS levels, primary hepatocytes (1 × 10^5^ cells/mL) were treated with tumor necrosis factor (TNF)-α (100 ng/mL), ZC (10 μM) and Mito-TEMPO (50 μM) for 24 h. To detect lipid accumulation, primary hepatocytes (1 × 10^5^ cells/mL) were reacted with TNF-α (100 ng/mL), ZC (10 μM) and Mito-TEMPO (50 μM) for 24 h, followed by BODIPY staining.

### 4.5. Short Interfering RNA (siRNA) to Knock down Gene Expression

Primary hepatocytes were cultured in 12-well plates for 4 h and transfected with control siRNA or TNFR1 siRNA (Integrated DNA Technologies, Inc., Coralville, IA, USA) using Lipofectamine 3000 (Thermo Fisher Scientific, Waltham, MA, USA). During the transfection period of 24 h, TNF-α was co-treated and used for qRT-PCR analysis and mitochondrial superoxide (MitoSOX) measurement.

### 4.6. Cytotoxicity Assay

ImKCs and Hep3B cells were plated on a 12-well plate (2 × 10^5^ cells/mL) and cultured for 24 h at 37 °C using a medium supplemented with 10% fetal bovine serum (FBS) in Dulbecco’s Modified Eagle’s Medium (DMEM). After 24 h, a medium treated with ZC at different concentrations (2.5, 5 and 10 μM) was collected in a medium that did not contain fetal bovine serum (FBS), and cytotoxicity was evaluated. Cytotoxicity was performed using the Quanti-LDH™ Cytotoxicity Assay Kit (BioMax, Guri-si, Republic of Korea, BCT-LDH1000).

Primary hepatocytes were isolated and cultured in collagen-coated 12-well (2 × 10^5^ cells/mL) plates with M199 medium supplemented with 10% fetal bovine serum (FBS) and 1% antibiotic antimycotic (Gibco, Grand Island, NY, USA). After 4 h, the medium was replaced with a medium supplemented with only 1% antibiotic antimycotic (Gibco, NY, USA) without fetal bovine serum, and various concentrations of ZC (2.5, 5 and 10 μM) were added. After 24 h of incubation, the medium was collected and cytotoxicity was evaluated. Cytotoxicity was performed using a Quanti-LDH™ Cytotoxicity Assay Kit (BioMax, BCT-LDH1000).

### 4.7. Enzyme-Linked Immunosorbent Assay (ELISA)

ImKCs (2 × 10^5^) treated with LPS (125 ng/mL) and ZC (10 μM) were cultured in a 12-well plate. After 6 h, the supernatant was tested for cytokine (TNF-α) secretion using a commercial enzyme immunoassay kit (R&D Systems, Minneapolis, MN, USA) according to the manufacture’s protocol.

### 4.8. Quantitative Real-Time Polymerase Chain Reaction (qRT-PCR) and Mitochondrial DNA Analysis

Cells were homogenized by treatment with RiboEX (Cat. No. 301-001), and Geneall’s Hybrid-R kit (Cat. No. 305-101) was used for RNA extraction. Total RNA was reverse transcribed into complementary DNA (cDNA) using a DNase (Promega, Madison, WI, USA, Cat. No. M6101) and High-Capacity cDNA Reverse Transcription Kit (Applied Biosystems™, Cat. No. 4368814). To perform PCR, CFX Connect Real-Time PCR Detection System (Bio-Rad, Hercules, CA, USA) was used. Target gene expression was normalized to glyceraldehyde 3-phosphate dehydrogenase (GAPDH) and β-Actin levels as internal controls. The specific primer sequences are listed in Table 1.

The quantification of mtDNA was performed as described [52]. Primary hepatocytes (2 × 10^5^) treated with TNF-α (100 ng/mL) and ZC (10 μM) were cultured in a 12-well plate. After 24 h, qRT-PCR was performed after DNA was extracted using a DNA purification kit (GeneAll^®^ Exgene™ Cell SV, Seoul, Republic of Korea) according to the manufacturer’s protocol. The specific primer sequences are listed in Table 1.

### 4.9. Western Blot Assay

The cells were homogenized by directly treating them with RIPA (radioimmunoprecipitation assay) buffer on ice for a duration of 30 min. Following homogenization, cell lysates were centrifuged at 13,000 rpm for 15 min. The protein concentrations within the lysates were determined using a Pierce BCA Protein Assay Kit, which was provided by Thermo Fisher Scientific Inc. located in Waltham, MA, USA. Subsequently, the supernatant from the lysate was subjected to separation on either a 10% or 12.5% SDS-PAGE (Sodium Dodecyl Sulfate–Polyacrylamide Gel Electrophoresis) gel and subsequently transferred to a PVDF (Polyvinylidene Fluoride) membrane. The PVDF membrane was then blocked by incubating it with 5% skim milk for 1 h at room temperature. Primary antibodies are NF-κB (ABclonal, A11201), phosphorylated-NF-κB (Cell signaling, #3033), phosphorylated-IκBα (Cell signaling, #2859), superoxide dismutase 1 (SOD1, ABclonal, A12537), β-Actin (Sigma, A5442) was diluted 1:1000 and incubated overnight at 4 °C. Then, the horseradish peroxidase-conjugated secondary antibody was diluted at room temperature for 1 h. Antibodies were diluted in tris-buffered saline/Tween containing 2% or 5% bovine serum albumin.

### 4.10. Measurement and Staining of Mitochondrial Superoxide (MitoSOX)

ImKCs (5 × 10^4^ cells/mL) and Hep3B cells (3 × 10^4^ cells/mL) were plated, and 10% FBS (fetal bovine serum) was added to Dulbecco’s Modified Eagle’s Medium (DMEM) at 37 °C for 24 h. After 24 h, ImKCs were treated with ZC and Mito-TEMPO for 6 h in a medium without FBS (fetal bovine serum) added, with or without LPS. In Hep3B cells, ZC and Mito-TEMPO were treated for 24 h in a medium without fetal bovine serum (FBS) added, with or without TNF-α. Cells were washed twice with phosphate-buffered saline (PBS) and stained with MitoSOX (Invitrogen, Waltham, MA, USA, M36008) reagent for 15 min. The cell was washed away with PBS, and the fluorescent expression value was measured using a microplate reader (TECAN, Spark). For cell staining, cells were cultured under the same conditions, stained with MitoSOX (Invitrogen, M36008) reagent for 15 min, washed with PBS, and fixed with 4% Paraformaldehyde (biosesang, PC2031-100-00) for 20 min. It was washed with PBS and stained with DAPI (diamidino-2-phenylindole) for 5 min. For the quantitative analysis of mtROS production, the scanned cells were captured using a DMi8 (Leica Camera, Wetzlar, Germany) at 400× magnification.

### 4.11. Staining of Neutral Lipid Droplets for Microscopy

Primary hepatocytes were isolated and cultured in collagen-coated 12-well (1 × 10^5^ cells/mL) plates with M199 medium supplemented with 10% fetal bovine serum (FBS) and 1% antibiotic antimycotic (Gibco, NY, USA). After 4 h, the medium was replaced with a medium supplemented with only 1% antibiotic antimycotic (Gibco, NY, USA) without fetal bovine serum. ZC, Mito-TEMPO was treated for 24 h. After 24 h of incubation, the cells were washed 3 times with phosphate-buffered saline (PBS) and fixed with 4% paraformaldehyde (biosesang, Yongin-si, Republic of Korea, PC2031-100-00) for 30 min. It was washed three times with PBS and stained with BODIPY and DAPI (diamidino-2-phenylindole) reagent for 20 min. Liver sections were washed three times with PBS, and scans for the quantitative analysis of hepatocellular lipid accumulation were captured using a DMi8 (Leica Camera, Wetzlar, Germany) at 400× magnification. BODIPY positive areas were measured using LAS X (Leica, Wetzlar, Germany) and ImageJ (NIH) software, 1.52V.

### 4.12. Flow Cytometry Analysis for Mitophagy

For the detection of mitophagy, we initiated the process by transfecting the Hep3B cells with the Mt-Keima plasmid. These transfected cells were then seeded into a 12-well cell culture plate at a density of approximately 80%. Following the desired treatment, we carefully washed the cells with PBS to remove any residual culture medium. Subsequently, we employed trypsin to effectively lyse the cells and obtain a cell pellet. This cell pellet served as the basis for our mitophagy analysis. To prepare the sample for Flow Cytometry, we resuspended the cell pellet in 200 μL of FACS buffer. This step ensured that the cells were appropriately suspended and ready for flow cytometry analysis. The Flow Cytometry analysis was conducted according to the predetermined mitophagy settings. This allowed us to measure and assess the degree of mitophagy within the Hep3B cells, based on the fluorescence signals from the Mt-Keima plasmid.

### 4.13. Statistical Analysis

All data were expressed as the mean ± standard error (sem) from at least 3 independent experiments. Statistical analyzes were performed using an unpaired student’s *t*-tests in GraphPad Prism 9 (GraphPad Software Inc. San Diego, CA, USA). A *p* value < 0.05 was considered statistically significant.

## 5. Conclusions

In conclusion, ZC showed antioxidant, anti-inflammatory and anti-steatotic effects in Kupffer cells (KCs) and hepatocytes, respectively. ZC suppressed mtROS-mediated NF-κB signaling in KCs and improved the inflammatory response. ZC inhibited TNF-α-induced mtROS production in hepatocytes and improved mtROS-mediated lipid accumulation by aiding the fatty acid oxidation process. Therefore, ZC can be used as a therapeutic strategy for NAFLD by modulating mtROS in KCs and hepatocytes to improve inflammation and hepatic steatosis.

## Figures and Tables

**Table 1:** Primer sequences of the genes used for quantitative real-time polymerase chain reaction.

Gene.	Forward (5′ to 3′)	Reverse (5′ to 3′)
**Mouse**		
GAPDH	ACGGCAAATTCAACGGCACAG	AGACTCCACGACATACTCAGCAC
IL-1β	CTCGCAGCAGCACATCAACAAG	CCACGGGAAAGACACAGGTAGC
TNF-α	AGGGTCTGGGCCATAGAACT	CCACCACGCTCTTCTGTCTA
CCL2	ATTGGGATCATCTTGCTGGT	CCTGCTGTTCACAGTTGCC
CCL3	TCCCAGCCAGGTGTCATTTTCC	CAGTTCCAGGTCAGTGATGTATTCTTG
CCL4	CTCTGCGTGTCTGCCCTCTC	TGGTCTCATAGTAATCCATCAC
CXCL2	GCCAAGGGTTGACTTCAAGAACA	AGGCTCCTCCTTTCCAGGTCA
CXCL9	GGAACCCTAGTGATAAGGAATGCA	TGAGGTCTTTGAGGGATTTGTAGTG
TNFR1	GCTGGAGATGCAGAACGGGC	ACGAGGGGGCGGGATTTCTC
16sRNA	CCGCAAGGGAAAGATGAAAGAC	TCGTTTGGTTTCGGGGTTTC
HK2	GCCAGCCTCTCCTGATTTTAGTGT	GGGAACACAAAAGACCTCTTCTGG
**Human**		
β-Actin	AGAGCTACGAGCTGCCTGAC	AGCACTGTGTTGGCGTACAG
CPT1	TCCAGTTGGCTTATCGTGGTG	TCCAGAGTCCGATTGATTTTTGC
ACADM	GCCTGCTTGTTGCTGTAGACTCC	ATTGCTGCCTGATTGTTCCTCTGG
HADHA	TTACCAGGAGCAGGAGGCACAC	GAGGCAGGAGAATCGCTTGAACC
PGC-1α	TTTGTTCCTTGCTTCTTTGGCTTCTTG	TGCTTGGCTACGGTAATCATCACTATG
TFAM	CTCGCTTCTCCCATCCCTCCTC	GTATCCAACGCCTTCAACAAGAATGC
Nrf1	CCAGACGACGCAAGCATCAGAG	CTGTTCCAATGTCACCACCTCCAC
Nqo-1	GCTGGAGTGCAGTGGTGTGATC	ACTTTGGGAGGCTGAGGTAGGC
Acox1	GGCGAGACCTCCCTCCTTTCC	CAGGGTCAGCGATGCCAAACTC
Ucp2	GGCTGGAGGTGGTCGGAGATAC	GGCAGAAGTGAAGTGGCAAGGG

GAPDH, glyceraldehyde 3-phosphate dehydrogenase; TNF, tumor necrosis factor; IL, interleukin; CCL, C-C motif ligand; CXCL, C-X-C motif ligand; CPT-1, carnitine palmitoyltransferase I; ACADM, acyl-Coenzyme A dehydrogenase; HADHA, hydroxyacyl-CoA dehydrogenase trifunctional multienzyme complex subunit alpha; PGC-1α, peroxisome proliferator-activated receptor gamma coactivator 1-alpha; TFAM, mitochondrial transcription factor A; Nrf1, nuclear respiratory factor 1; Nqo-1, NAD(P)H quinone dehydrogenase 1; Acox1, acyl-CoA oxidase 1; Ucp2, uncoupling protein 2.

## Data Availability

Data is contained within the article.

## Abbreviations

NAFLD	Non-alcoholic fatty liver disease
KCs	Kupffer cells
mtROS	Mitochondrial ROS
ZC	Zaluzanin C
SOD	Superoxide dismutases
MitoSOX	Mitochondrial superoxides
ImKC	Immortalized mouse kupffer cells
LPS	Lipopolysaccharide
TNF	Tumor necrosis factor
CCL	C-C motif ligand
CXCL	C-X-C motif ligand
CPT-1	Carnitine palmitoyltransferase I
ACADM	Acyl-Coenzyme A dehydrogenase
HADHA	Hydroxyacyl-CoA dehydrogenase trifunctional multienzyme complex subunit alpha
PGC-1α	Peroxisome proliferator-activated receptor gamma coactivator 1-alpha
TFAM	Mitochondrial transcription factor A
Nrf1	Nuclear respiratory factor 1
Nqo-1	NAD(P)H quinone dehydrogenase 1
Acox1	Acyl-CoA oxidase 1
Ucp2	Uncoupling protein 2
